# Sex hormones influence survival of patients with metastatic urothelial carcinoma undergoing immune checkpoint therapy

**DOI:** 10.1186/s13293-023-00522-x

**Published:** 2023-06-05

**Authors:** Andrea Katharina Lindner, Felizian Lackner, Piotr Tymoszuk, Dominik Andreas Barth, Andreas Seeber, Florian Kocher, Bettina Toth, Margarethe Hochleitner, Martin Pichler, Renate Pichler

**Affiliations:** 1 Department of Urology, Comprehensive Cancer Center Innsbruck, Medical University of Innsbruck, Anichstrasse 35, 6020 Innsbruck, Austria; 2 Data Analytics As a Service Tirol, Innsbruck, Austria; 3 Division of Oncology, Department of Internal Medicine, Medical University of Graz, Graz, Austria; 4 Department of Hematology and Oncology, Comprehensive Cancer Center Innsbruck, Medical University of Innsbruck, Innsbruck, Austria; 5 Department of Gynecological Endocrinology and Reproductive Medicine, Medical University Innsbruck, Innsbruck, Austria; 6 Department of Internal Medicine, Gender Medicine Unit, Medical University of Innsbruck, Innsbruck, Austria; 7 Translational Oncology, University Hospital of Augsburg, Augsburg, Germany

**Keywords:** Metastatic urothelial carcinoma, Immunotherapy, Overall survival, Sex hormones, Gonadotropins, Prognostic, Biomarkers

## Abstract

**Introduction:**

Clinical trials investigating efficacy of immune checkpoint inhibitors (ICI) revealed sex-specific divergent outcomes in urothelial cancer (UC), suggesting that sex hormones might play an important role in gender-specific dimorphisms of response upon ICI. However, further clinical investigations are still needed to understand the influence of sex hormones in UC. The aim of this study was to get further insights on the prognostic and predictive value of sex hormone levels in patients with metastatic UC (mUC) who underwent ICI.

**Material and methods:**

Sex hormone levels of patients with mUC including luteinizing hormone (LH), follicle-stimulating hormone (FSH), LH/FSH ratio, prolactin, testosterone and 17β-estradiol (E2) were evaluated at baseline and during ICI at 6/8 weeks and 12/14 weeks.

**Results:**

Twenty-eight patients (10 women, 18 men) with a median age of 70 years were included. Metastatic disease was confirmed in 21 patients (75%) after radical cystectomy while seven patients showed mUC at first diagnosis. Twelve patients (42.8%) received first line and 16 patients second line pembrolizumab. The objective response rate (ORR) was 39% (CR in 7%). The median progression-free survival (PFS) and overall survival (OS) was 5.5 and 20 months. Focusing on changes of sex hormone levels during ICI, a significant increase in FSH levels and decrease of the LH/FSH ratio was noticed in responders (*p* = 0.035), yet without sex-specific significance. When adjusted for sex and treatment line, a significant increase of FSH levels was confirmed in men during second line pembrolizumab. Focusing on baseline levels, LH/FSH ratio was significantly higher in female responders (*p* = 0.043) compared to non-responders. In women, increased LH levels and LH/FSH ratio were associated with better PFS (*p* = 0.014 for LH, *p* = 0.016 for LH/FSH ratio) and OS (*p* = 0.026 and *p* = 0.018). In male patients, increased E2 levels were linked with improved PFS (*p* < 0.001) and OS (*p* = 0.039).

**Conclusion:**

Increased LH and LH/FSH values in women as well as high E2 levels in men were significant predictors of better survival. Elevated LH/FSH ratio was predictive of better response to ICI in women. These results show first clinical evidence of the potential role of sex hormones as prognostic and predictive biomarker in mUC. Further prospective analyses are needed to corroborate our findings.

**Supplementary Information:**

The online version contains supplementary material available at 10.1186/s13293-023-00522-x.

## Plain language summary

Urothelial carcinoma (UC) presents as aggressive disease with a greater incidence in men, yet a more aggressive course of disease in women. Patients with metastatic UC receive a chemotherapy regimen as the gold standard, based on an included platin substance. In the case of having contraindications to chemotherapy, checkpoint immunotherapy, priming the immune system to the tumor, is the treatment of choice. Furthermore, immunotherapy is used as second line therapy in progressive disease after chemotherapy and as maintenance therapy in stable tumor conditions after completing the chemotherapy regimen.

Evidence shows that sex hormones of the hypothalamus–hypophysis axis influence development and course of UC. The sex hormones luteinizing hormone (LH) and follicle-stimulating hormone (FSH) stimulate estrogen (E2) production with a negative feedback function on the LH and FSH secretion. High levels of E2 present with a protective effect against UC. Sex has furthermore shown to predict potential response to immunotherapy. This study therefore focused on monitoring and correlating changes of sex hormone levels in 28 patients during therapy with checkpoint inhibitors.

This first study assessing changes in sex hormones and the influence of baseline sex hormone values on survival in UC shows that responders to immunotherapy had significantly increased FSH levels. FSH furthermore increased in male patients receiving second line immunotherapy. High values of LH and a high LH/FSH ratio at baseline correlated with better overall survival in female patients. High E2 levels were indicative of better survival in male patients. The study results represent first suggestive prognostic and predictive results to the response of immunotherapy in UC.

**Supplementary Information:**

The online version contains supplementary material available at 10.1186/s13293-023-00522-x.

## Highlights

- Increased LH and LH/FSH values in women as well as high E2 levels in men were significant predictors of better survival.
- Elevated LH/FSH ratio was predictive of better response to immunotherapy in women.
- First clinical evidence of the potential role of sex hormones as prognostic and predictive biomarkers in metastatic urothelial cancer.

**Supplementary Information:**

The online version contains supplementary material available at 10.1186/s13293-023-00522-x.

## Introduction

Urothelial cancer (UC) is one of the best-known cancer dignities exhibiting considerable sex-specific differences. Male sex is associated with a greater incidence of up to 5-times compared to females. However, women are more often diagnosed in a more advanced and aggressive disease stage [1–4]. Primary metastatic disease in UC accounts for approximately 5% of all newly diagnosed cases with a poor 5-year survival of only 5% [5]. According to international guidelines, aside from anatomical differences, female and male UC patients are clinically managed in the same way [6, 7]. Of note, a recent systematic review showed that female patients with muscle-invasive bladder cancer have a significantly increased risk of worse overall survival (OS) compared to male individuals [8].

Standard of care for first line treatment of metastatic UC (mUC) is a cisplatin-based chemotherapy combination [7]. Up to 50% remain ‘cisplatin-unfit’ by definition, which lead to implementation of a chemotherapy-regimen with carboplatin in these patients [9]. On the basis of two phase II clinical trials, two checkpoint inhibitors (ICI), namely the PD-1 receptor inhibitor pembrolizumab [10] and the PD-L1 inhibitor atezolizumab [11], have been approved by the FDA and EMA as the first line treatment in cisplatin-unfit mUC patients [10, 11].

Pembrolizumab demonstrated a significant OS improvement in patients with progressive disease after receiving first line platin-based chemotherapy [12]. On the basis of its high efficacy, pembrolizumab is currently recommended in international guidelines as second line therapy in mUC [7]. In patients with stable or remissive mUC after first line cisplatin-based chemotherapy, avelumab, a PD-L1 inhibitor is moreover approved as further maintenance therapy [13]. Resistance to standard therapies and high aggressiveness of mUC require a clinical shift towards individualized medicine, thus implying the need for further research in the field of sex-specific survival.

Several lines of evidence suggest a role of sex steroid hormones in UC, yet detailed mechanisms behind this remain unclarified. The sex hormones luteinizing hormone (LH) and follicle-stimulating hormone (FSH) are members of the gonadotropin family and secreted by the anterior pituitary gland. Both stimulate gonadal 17β-estradiol (E2) production with a negative feedback function on the pituitary LH and FSH secretion [14]. Literature indicates the potential role of E2 and androgens influencing the development and course of UC [15]. McGraph et al. described an increased risk of UC in postmenopausal women compared with premenopausal women, irrespectively of an experienced natural or surgical menopause [16]. This fact suggests E2 to significantly influence tumor physiology.

In addition, expression of the androgen receptor (AR) was repeatedly correlated with carcinogenesis [17, 18], accounting for the accumulation of male disease incidence. Loss of AR responsiveness has shown to lead to disease progression via androgen-independent pathways, maybe explaining the higher rates of progression in women [19, 20]. Given the common embryological origin of the bladder trigone and the upper portion of the vagina, there is presence of estrogen receptor expression in the female bladder. E2 seems to have a protective effect against bladder tumorigenesis via the estrogen receptor pathway [21]. Furthermore, female patients receiving E2 replacement therapy are suggested to have a reduced UC risk [22, 23].

Sex is also considered for being a responsible factor influencing the patients individual immune and immunotherapeutic response [21]. Moreover, sex has shown potential to predict response to PD-1/PD-L1 targeting ICI, presenting poorer outcomes in women [24]. PD-L1 and other immune checkpoints are likely to be implicated in the female bladder, which is exposed to higher estrogen levels than the male bladder [25].

We recently showed that sex hormones of the pituitary–gonadal axis have already shown to be accountable for differences in responses to checkpoint-based therapies in renal cell carcinoma [26], yet, to date, the influence of sex hormones on response of ICI in UC has not been described. Therefore, the aim of this study was for the first time to monitor and correlate changes of sex hormone levels during therapy with checkpoint inhibitors in mUC with focus on therapy response. Additionally, we evaluated if baseline levels of sex hormones could predict therapy response and survival.

## Materials and methods

This is a retrospective observational study based on an Austrian uro-oncology cancer database. Consent of the local ethics commission of the Medical University Innsbruck was obtained with the study approval number 1006/2017. Research work was performed in accordance with the 1964 Helsinki Declaration, its later amendments and institutional ethical standards based on good clinical practice [27].

### Patient population and data collection

Medical records of patients diagnosed with mUC and treated with the PD-1 inhibitor pembrolizumab in first and second line were reviewed retrospectively. All patients with a confirmed histopathology of mUC of the bladder, with no received hormone replacement therapy and eligible follow-up (FU) data at our outpatient department were included. Patients with no definitive histopathology, simultaneous other oncological disease, ongoing hormonal replacement therapy and those who were followed up elsewhere were excluded. Tumor classification was performed according to the 2016 tumor-node-metastasis (TNM) classification [28] and grading referring to the World Health Organization and International Society of Urological Pathology categorization [29]. Demographic patient data as well as tumor characteristics, sequence of systemic therapy, therapy response to PD-1 inhibition, dates of progression and time of death or last FU visit were collected. Pembrolizumab was administered as approved, intravenously at a fixed dosage of 200 mg once every three weeks [10, 12]. Since April 2020, the fixed dosage of 400 mg once every 6 weeks was additionally authorized.

### Follow-up and blood sampling

**Fig. 1:**
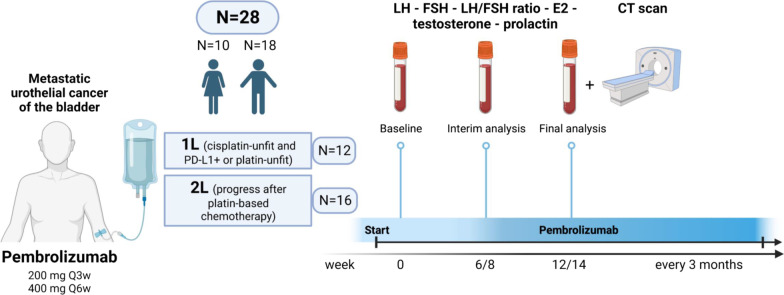
Flowchart of the study with patient recruitment and timing of the sample collection

FU visits were scheduled every 3 months and consisted of blood analysis and radiographic imaging. Imaging consisted of a chest and abdominopelvic computer tomography scan with contrast medium and urographic phase. Analysis of sex hormones including LH, FSH, LH/FSH ratio, E2, testosterone and prolactin were performed from the patient’s serum with enzyme-linked immunosorbent assays at three different points in time defined as: baseline at the beginning of immunotherapy in week 0, interim analysis after week 6/8 and final analysis in week 12/14. An overview of the sampling process is shown in Fig. 1. Reference values for female patients were 0.8–7.6 U/L for LH, 1.6–20.4 U/L for FSH, 11–43 ng/L for E2, 1.70–4.90 µg/L for testosterone and 2.5–17.0 µg/L for prolactin. In male patients, reference levels were 0.8–7.6 U/L for LH, 1.6–20.4 U/L for FSH, 11–43 ng/L for E2, 1.70–4.90 µg/L for testosterone and 2.5–17.0 µg/L for prolactin. Complete remission (CR), partial remission (PR) or stable disease (SD) in CT imaging as well as good tolerance of therapy were necessary for continuation of treatment. Progressive disease (PD) lead to a further therapy regimen in line. ORR was defined as the number of patients with response divided by the total number of patients. OS was defined as time from first therapy administration to death from all causes. Progression-free survival was specified as time from therapy begin to tumor progression, treatment discontinuation or tumor-specific death. In patients who have not progressed and still alive, the last time of FU was used as endpoint in time.

### Statistical analysis

Numeric features of the study cohort were presented as medians with interquartile ranges. Categorical variables were shown as percentages and total numbers within the set of complete observations. Normality of distribution of the hormone levels as well as normality of the individual differences in hormone levels between consecutive time points was investigated by the Shapiro–Wilk test and visual inspection of quantile–quantile plots. Changes in hormone levels were investigated by the Friedman test, for which patients were subdivided into two groups: stable disease/progressive disease (SD/PD) and complete remission/partial remission (CR/PR). Baseline hormone levels were compared using the Mann–Whitney test. Significance of differences in survival between hormone strata were assessed by Mantel–Haenszel test. The variable normality was checked separately for females and males. Statistical data analysis was performed with R (version 4.2.0), basic data transformation and re-coding tasks were done with the *tidyverse* package bundle and the *rlang* metaprogramming tool set. Numeric results were visualized with the *ggplot* and ExDA packages. Survival data were presented in Kaplan–Meier plots generated with the *survminer* and kmOptimizer packages. Report figures were built with the *cowplot* and figure packages. Report tables were created with the flextable package. To examine the sex hormone status and its associations with survival, patients were split into a high and low group using the gender-specific median as cutoff level. Overall response rate was defined as the number of patients with complete response or partial response divided by the total number of patients.

## Results

### Patient and tumor characteristics

Twenty-eight patients who were diagnosed and treated with mUC at our institution were included. Of these, 18 (64%) were male and 10 (36%) were female with a median age of 70 years (range 46–85). Age at diagnosis did not show any significance in baseline hormone values. 50% (*n* = 14) of all included patients had a smoking history and 75% (*n* = 21) underwent primary surgery after diagnosis according to the current recommended guidelines [7]. Histopathology revealed pure UC in all cases. In total, 3 (11%), 12 (43%), 4 (14%), 4 (14%), 3 (11%) and 2 (7.1%) patients presented with the tumors staged pT1, pT2a, pT2b, pT3a, pT3b and pT4a, respectively. Tumor grading resulted in 3.6% (*n* = 1) grade 2 and 96.4% (*n* = 27) grade 3. Thirteen (46%) patients had lymph node metastasis at diagnosis and seven (25%) patients presented in a metastatic disease. Twelve (42.8%) patients received pembrolizumab as first line therapy while 16 patients received it as second line therapy. No patient received ICI in any later therapy lines. Overall, the objective response rate (ORR) was 39% (*n* = 11) and 86% (*n* = 24) suffered disease progression in the study setting. Seventeen (61%) patients died before the date of completion of the study. Median duration of OS and progression-free survival (PFS) were 20 (range: 1–82) and 5.5 (range: 1–65) months.

### Sex hormone level characteristics during immunotherapy

**Fig. 2:**
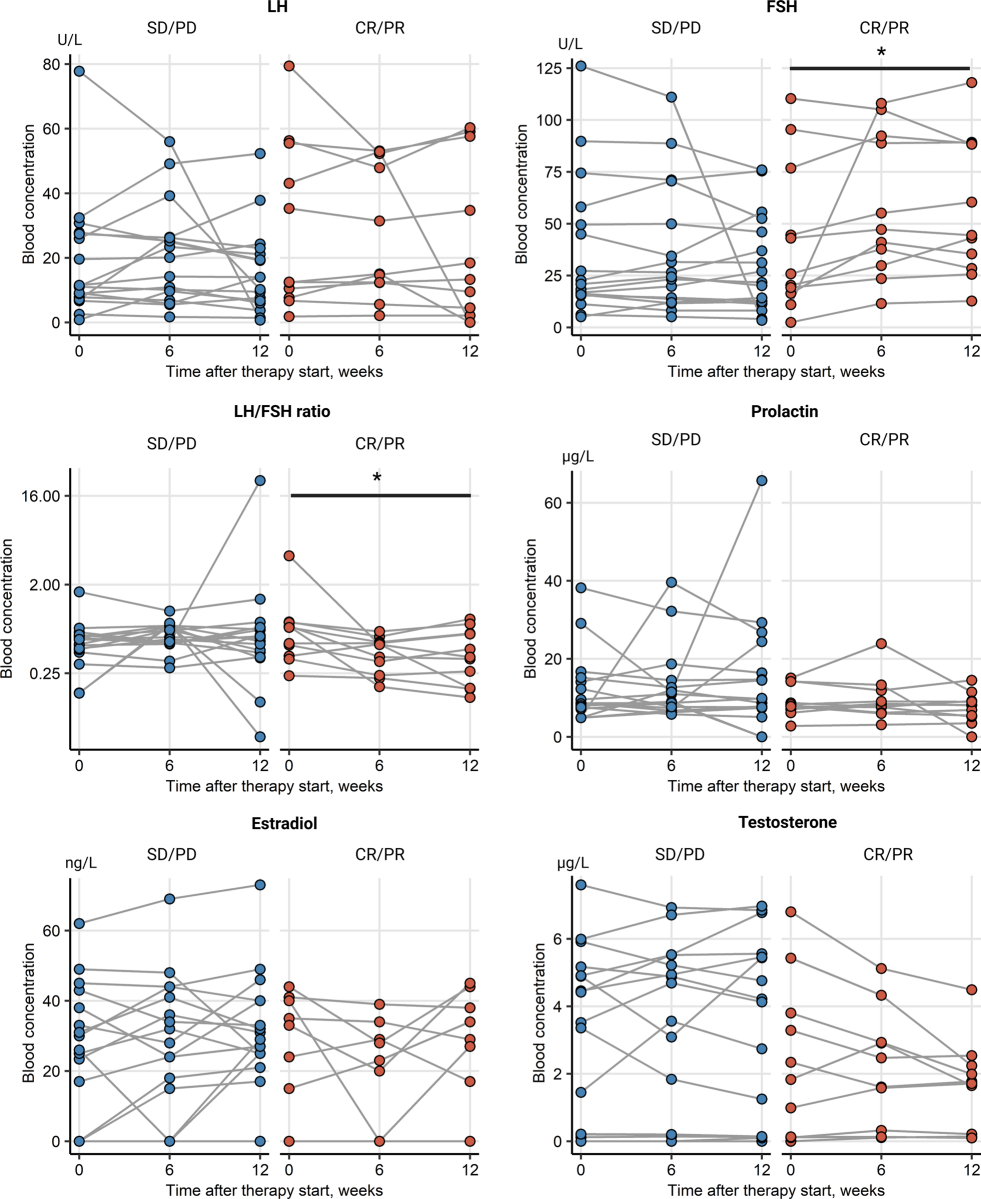
Overall changes of hormone levels during therapy regarding therapy outcome showing a significant rise in circulating FSH levels in patients with complete or partial response and a significant decrease of the LH/FSH ratio in the responder subset, *p < 0.05. SD/PD (*n* = 17), CR/PR (*n* = 11)

**Fig. 3:**
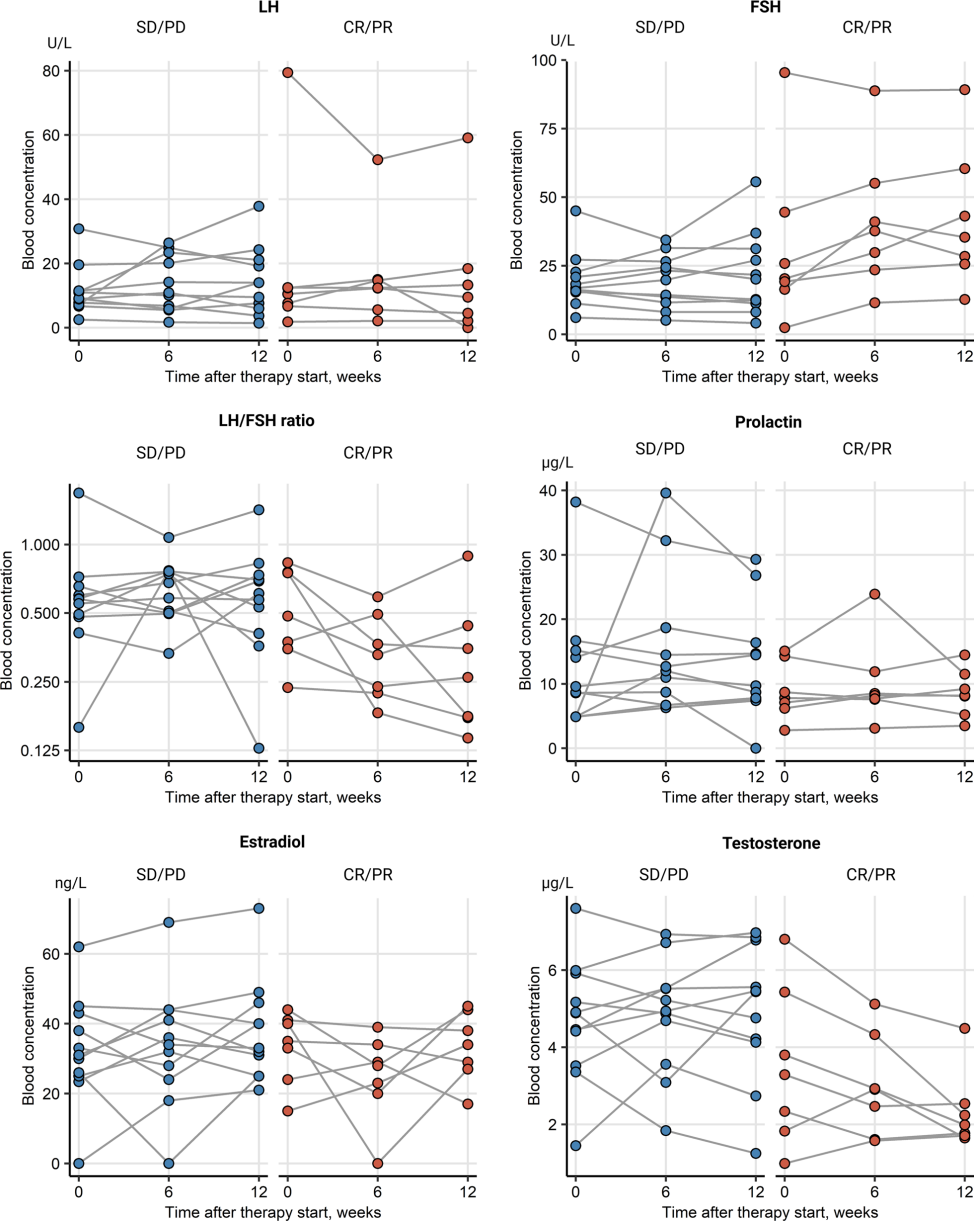
Changes of hormone levels during therapy in males regarding therapy outcome. Male therapy responders showed a, barely missed significant, rise in FSH concentrations, paralleled by a non-significant but noticeable drop of testosterone levels. SD/PD (*n* = 11), CR/PR (*n* = 7)

**Fig. 4:**
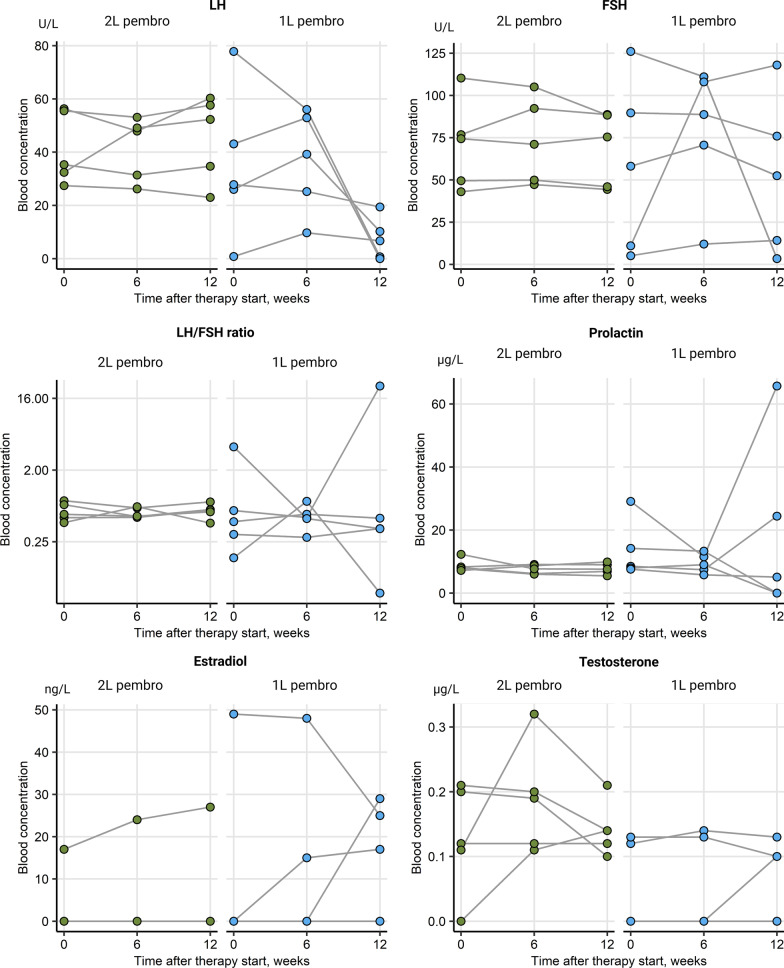
Changes of hormone levels during therapy in females with first and second-line immunotherapy. Females receiving first-line immunotherapy showed decreasing LH levels, just missing statistical significance. First line (1L) pembrolizumab (*n* = 5), second line (2L) pembrolizumab (*n* = 5)

**Fig. 5:**
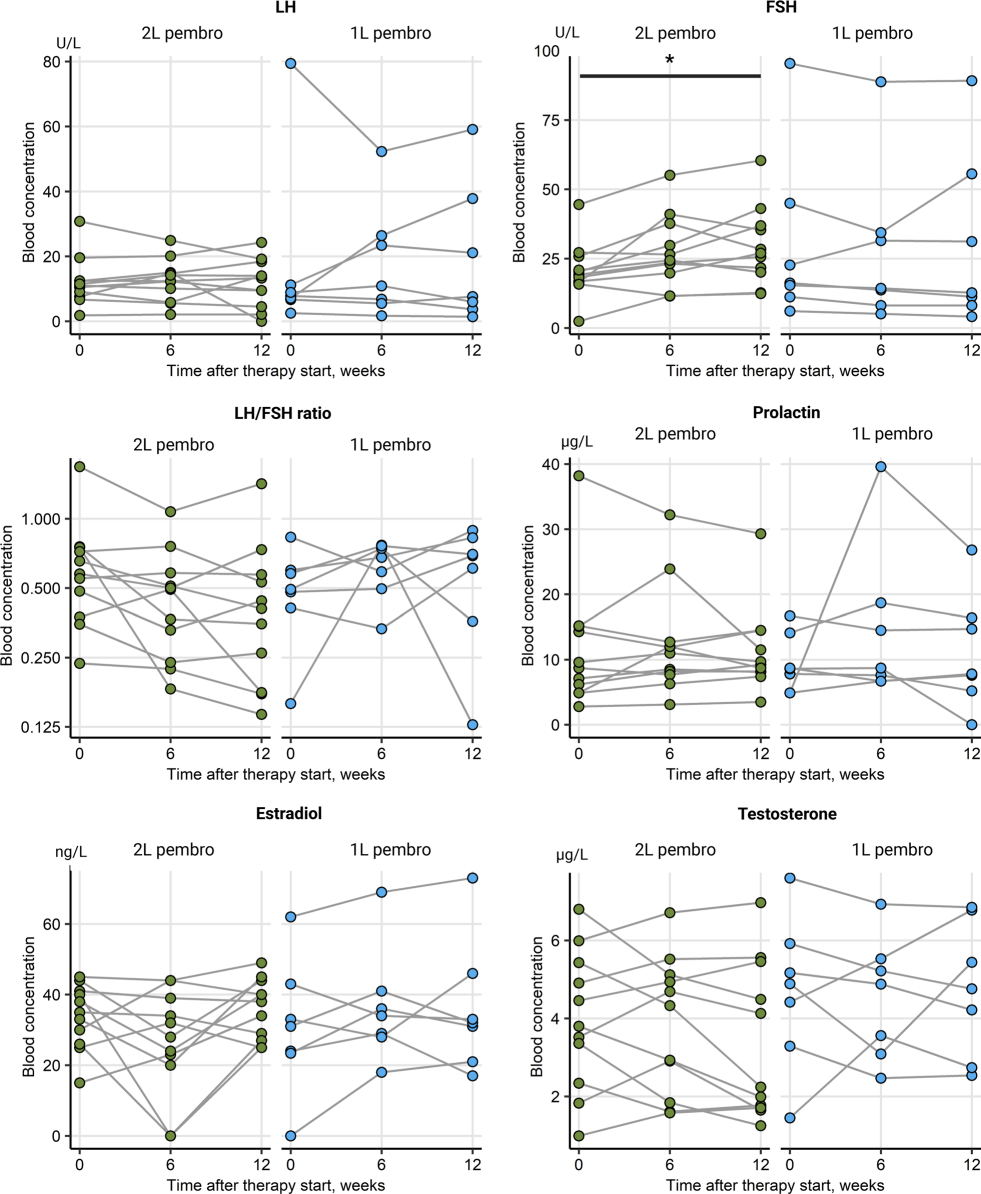
Changes of hormone levels during therapy in males with first and second-line immunotherapy. FSH levels significantly increased in men in second-line immunotherapy, *p < 0.05. 1L pembrolizumab (*n* = 7), 2L pembrolizumab (*n* = 11)

Change of levels of sex hormone levels including LH, FSH, LH/FSH ratio, prolactin, E2 and testosterone were collected during ICI for the whole patient cohort and analyzed separately for female and male patients. Alterations of the median blood hormone concentrations at baseline and during therapy were not significant in the entire cohort, as seen in Table 1. Additionally, there were no significant sex-specific deviations in hormone levels between time points, Additional file 1: Figure S1. An analogical analysis in therapy responders *vs.* non-responders, irrespective of gender, revealed a significant rise in circulating FSH levels in patients with complete or partial response (*p* = 0.035). This was paralleled by a significant decrease of the LH/FSH ratio in the responder subset (*p* = 0.035), Fig. 2. There were no differences in hormone levels between female responders and non-responders, yet a barely missed significant rise in FSH concentrations (*p* = 0.05) paralleled by a noticeable but non-significant drop of testosterone in male responders, Fig. 3. No significant changes of prolactin levels were observed in the female as well as in the male population. In females receiving first line ICI, only LH levels tended to decrease, yet just missing statistical significance (*p* = 0.074), Fig. 4. FSH levels significantly increased in men in second line ICI (*p* = 0.029), Fig. 5**.**

**Table 1:** Alterations of the median blood hormone concentrations at baseline and during therapy

Variable	Baseline	6/8 weeks	12/14 weeks	*p* value*	Effect size*
Participants, *n*	28	28	28		
LH (U/L)	12 [IQR: 7.8–31] range: 0.8–79	18 [IQR: 10–33] range: 1.7–56	14 [IQR: 5.6–23] range: 0–60	0.72	W = 0.012
FSH (U/L)	22 [IQR: 16–52] range: 2.4–130	33 [IQR: 18–71] range: 5.1–110	33 [IQR: 14–57] range: 3.4–120	0.6	W = 0.018
LH/FSH ratio	0.55 [IQR: 0.43–0.72] range: 0.16–3.9	0.51 [IQR: 0.46–0.68] range: 0.18–1.1	0.51 [IQR: 0.36–0.69] range: 0.056–23	0.65	W = 0.015
Prolactin (µg/L)	8.4 [IQR: 7.2–14] range: 2.8–38	8.7 [IQR: 7.2–12] range: 3.1–40	8.4 [IQR: 6.6–14] range: 0–66	0.94	W = 0.0023
Estrogen (ng/L)	26 [IQR: 0–38] range: 0–62	24 [IQR: 0–34] range: 0–69	28 [IQR: 17–38] range: 0–73	0.41	W = 0.032
Testosterone (µg/L)	2.8 [IQR: 0.13–4.9] range: 0–7.6	2.7 [IQR: 0.18–4.9] range: 0–6.9	1.9 [IQR: 0.14–4.6] range: 0–7	0.75	W = 0.01

*Friedman test with Kendall W effect size statistic

### Higher baseline LH/FSH ratio in female immunotherapy responders

**Fig. 6:**
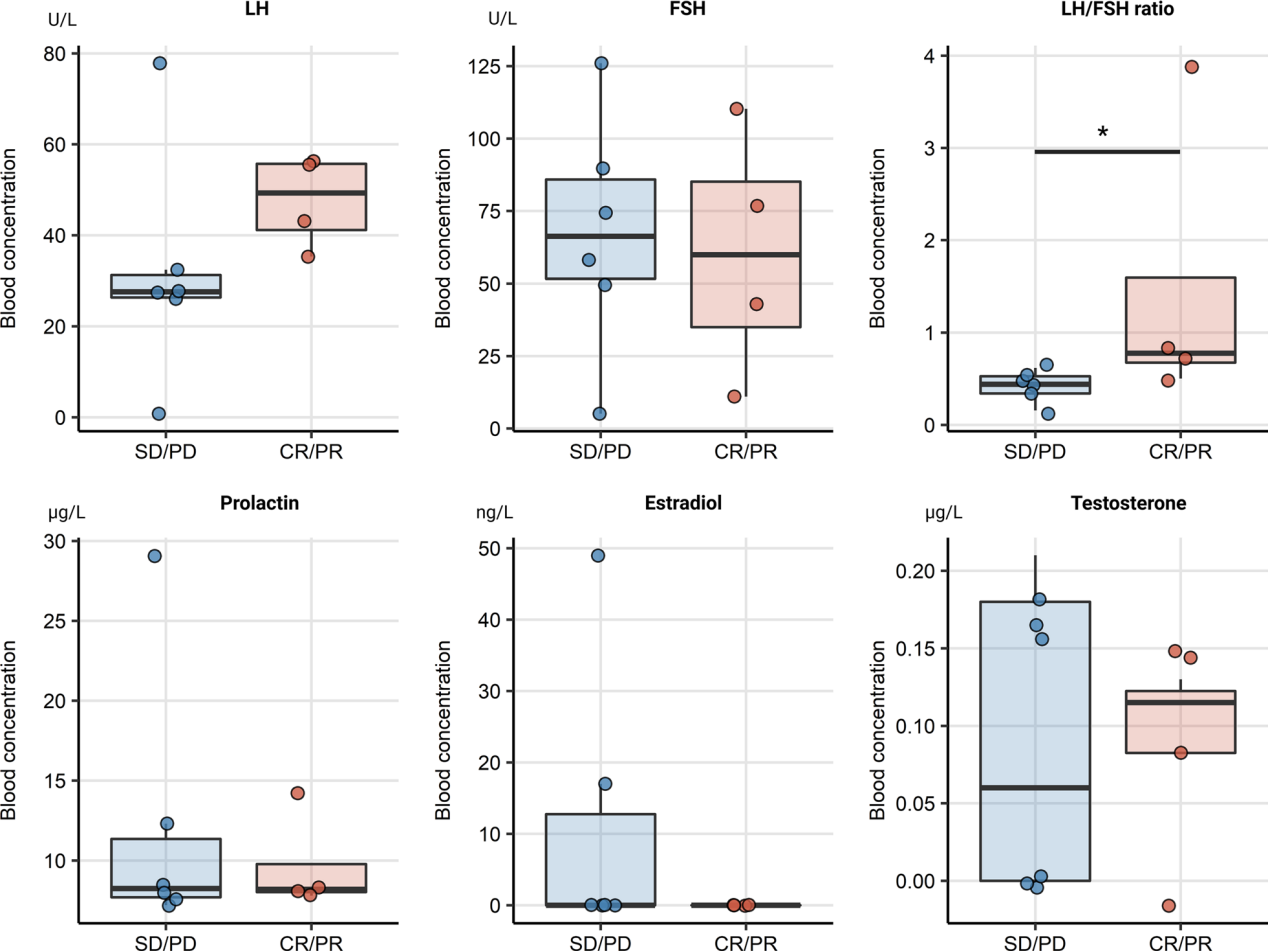
Baseline levels of sex hormones in female responders and non-responders. Boxes denote medians with interquartile ranges, whiskers span over 150% interquartile range. Female responders had a significantly higher LH/FHS ratio at baseline in therapy response, *p < 0.05. SD/PD (*n* = 6), CR/PR (*n* = 4)

**Fig. 7:**
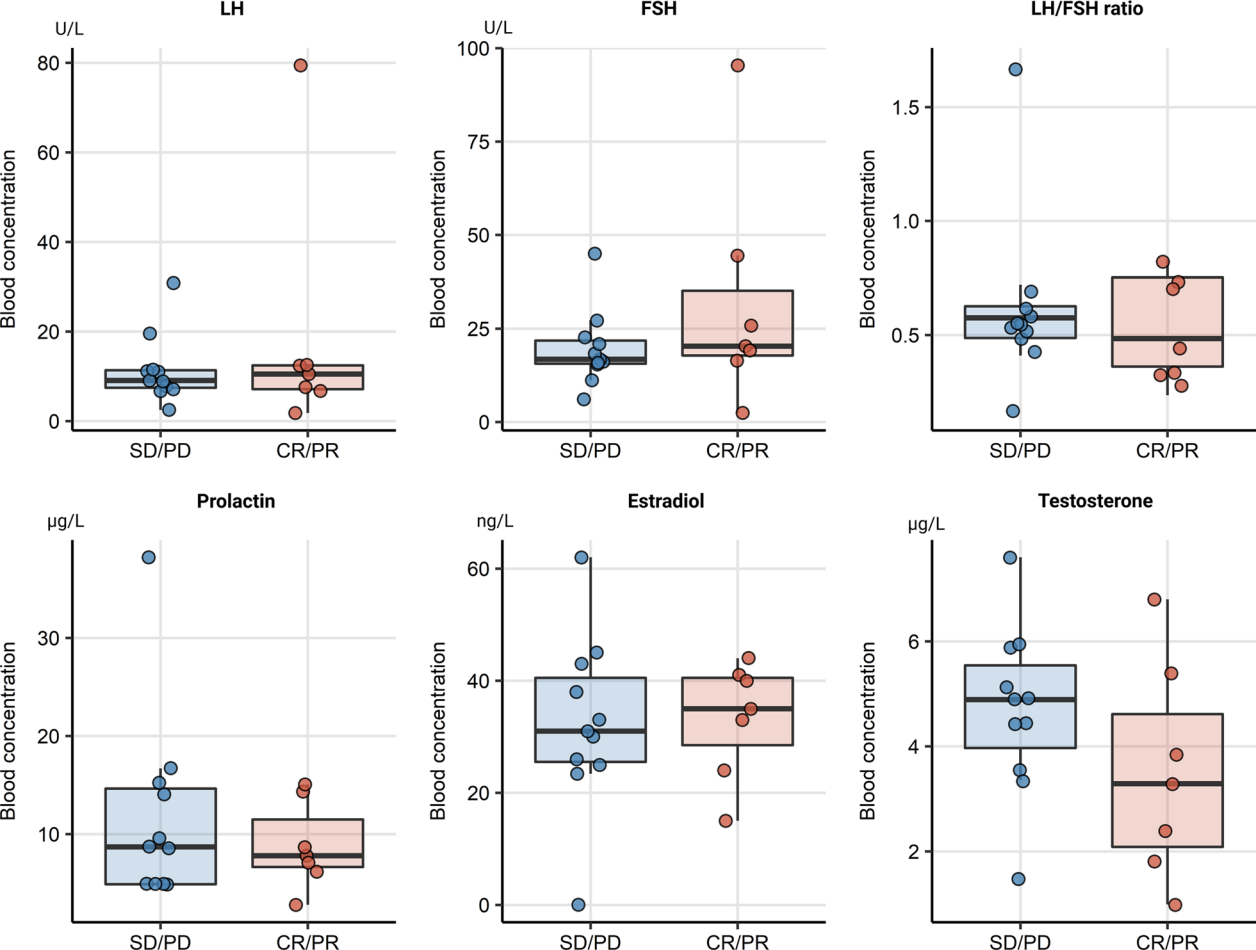
Baseline levels of sex hormones in male responders and non-responders. Boxes denote medians with interquartile ranges, whiskers span over 150% interquartile range. A non-significant trend towards lower testosterone levels was observed. SD/PD (*n* = 11), CR/PR (*n* = 7)

Comparison of baseline hormone concentrations between females with an overall therapy response (CR/PR) and without response (SD/PD) revealed a significantly higher LH/FSH ratio in responders (*p* = 0.043), as seen in Fig. 6. In male responders a direct trend towards lower testosterone levels could be noticed, this effect was however not significant, Fig. 7.

### High LH in females and high E2 in males correlate with better survival

**Fig. 8:**
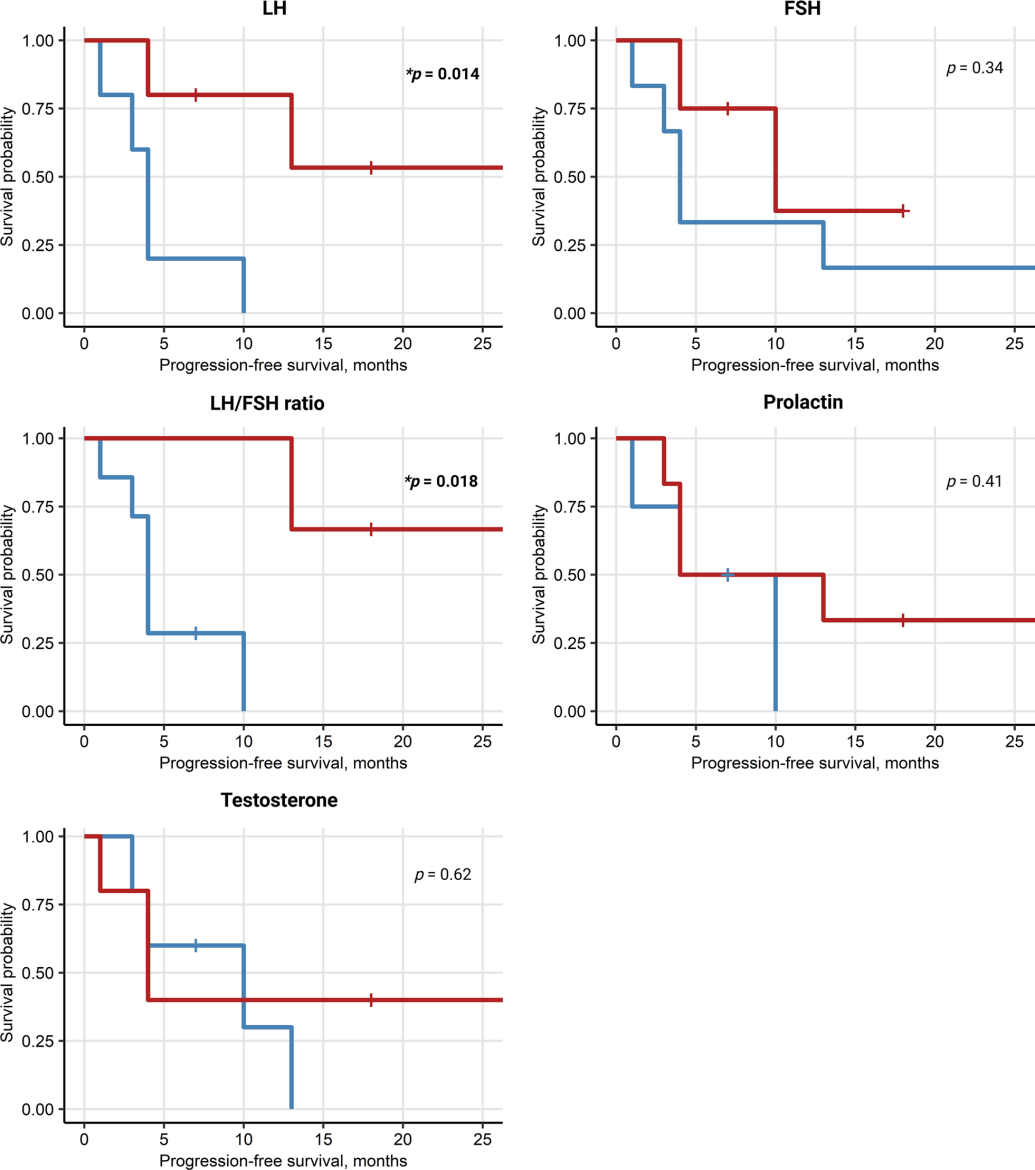
Progression-free survival (PFS) in females stratified by baseline sex hormone levels

**Fig. 9:**
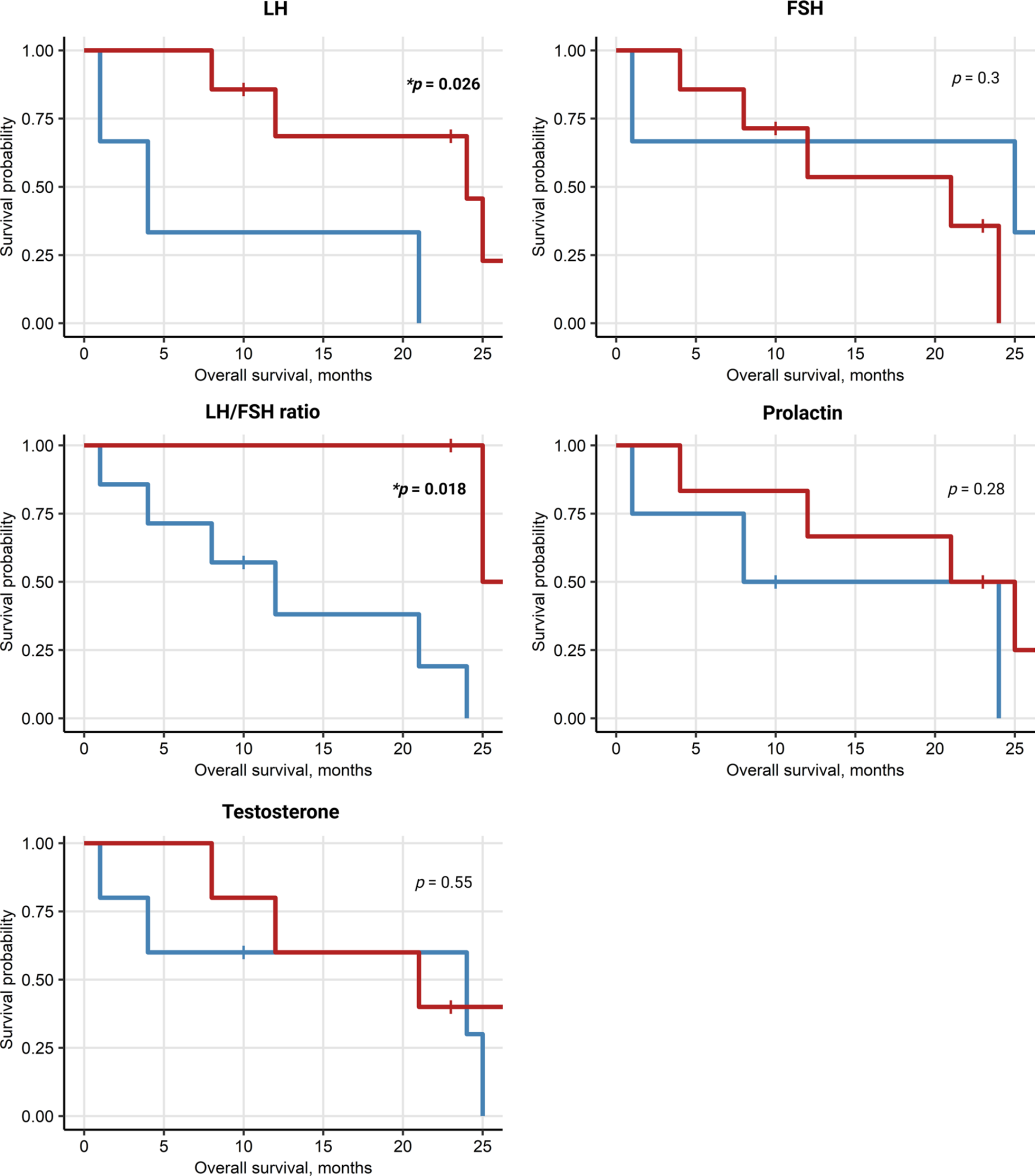
Overall survival (OS) in females stratified by baseline sex hormone levels

**Fig. 10:**
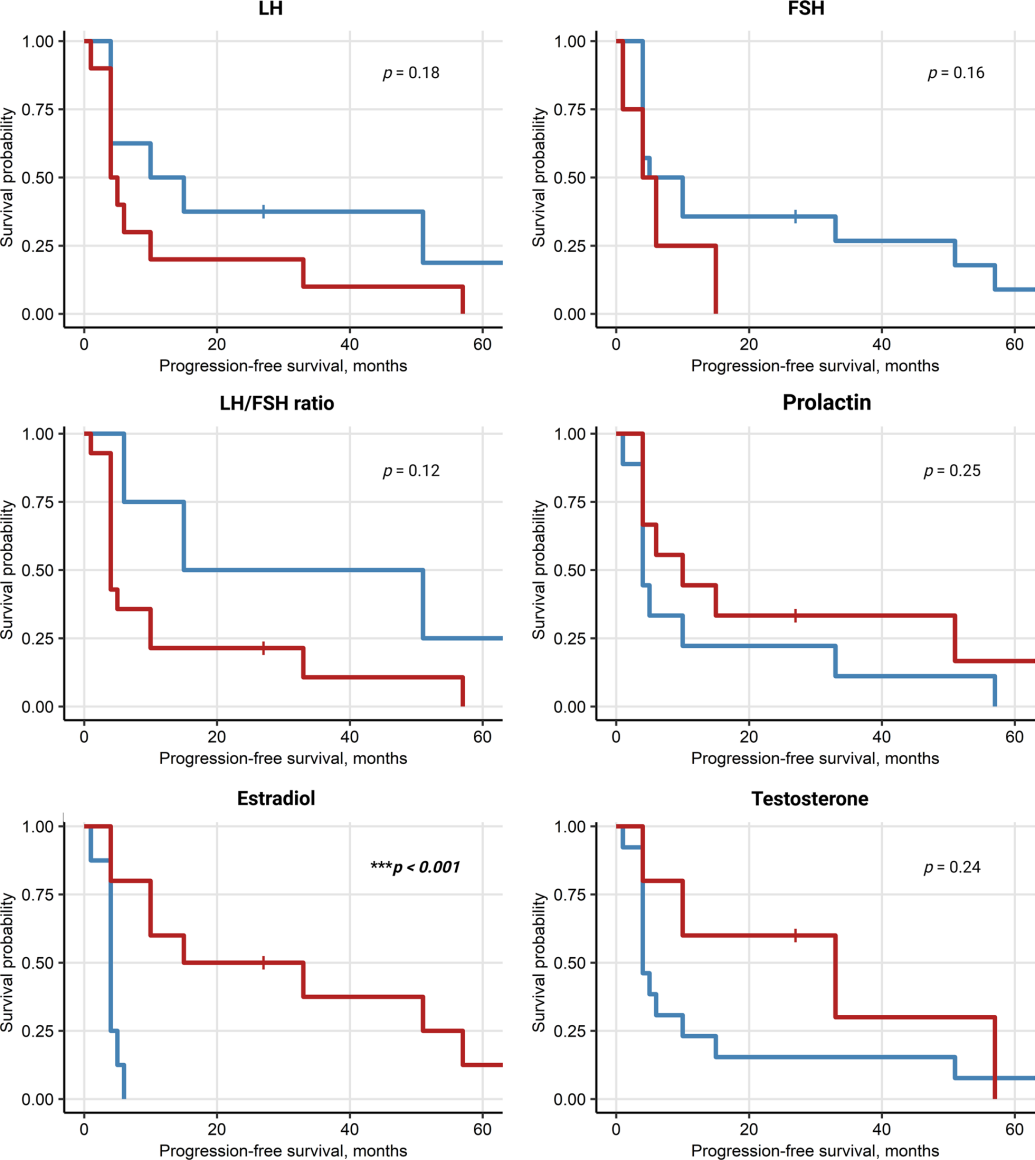
Progression-free survival (PFS) in males stratified by sex hormone levels

**Fig. 11:**
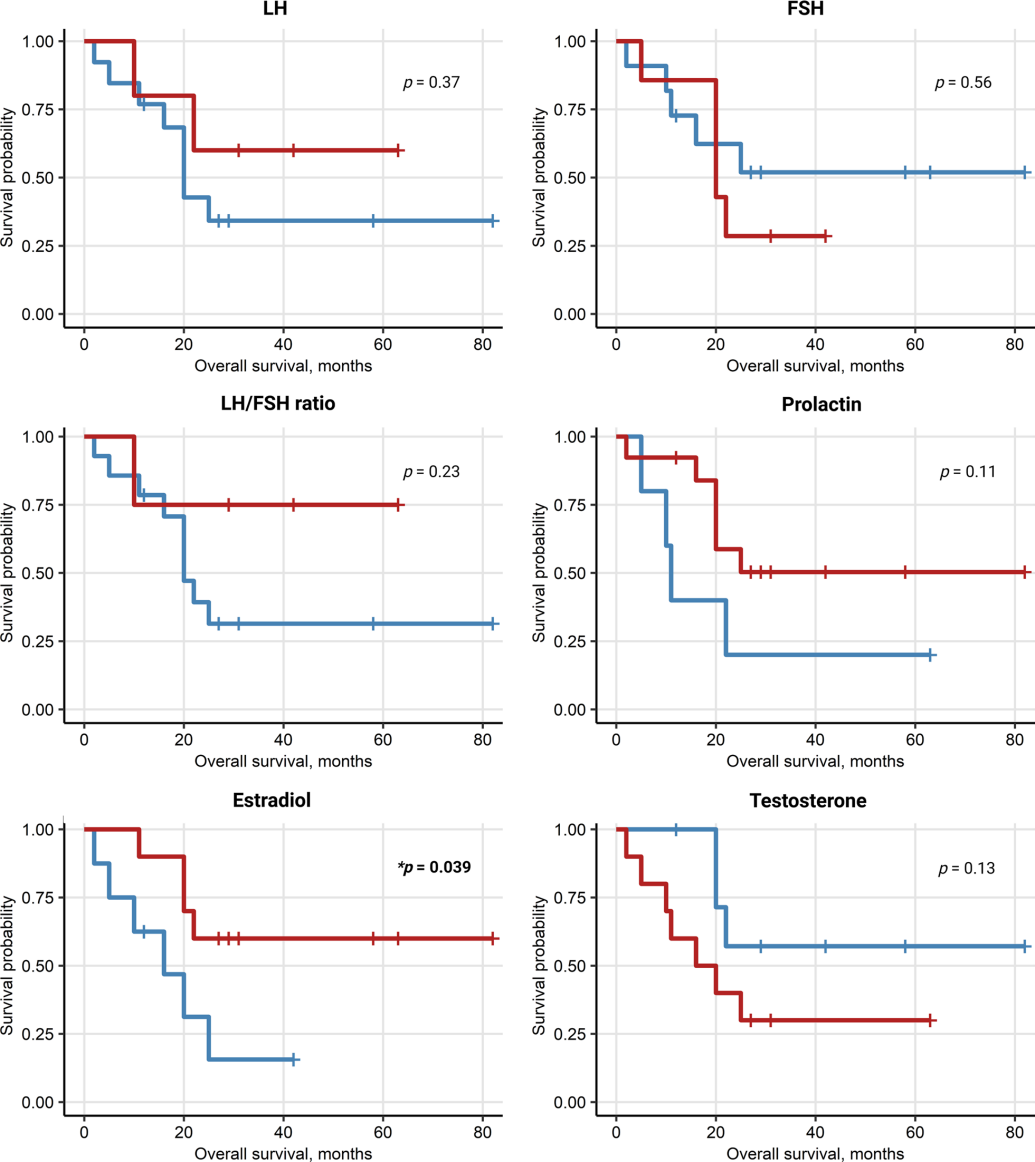
Overall survival (OS) in males stratified by sex hormone levels

We analyzed potential sex-specific correlations between sex hormone levels assessed at baseline with response to ICI (CR/PR) or outcomes (PFS, OS). In women, high levels of LH at baseline were found to be significantly correlated with better PFS (*p* = 0.014) and OS (*p* = 0.026). An even more significant association was seen between an increased LH/FSH ratio an PFS (*p* = 0.018) as well as OS (*p* = 0.018), Figs. 8, 9. In male patients, higher baseline levels of estrogen correlated with better PFS (*p* ≤ 0.001) and OS (*p* = 0.039), Figs. 10, 11. No other endocrine parameters correlated with PFS or OS in either female or male patients.

## Discussion

Sex differences with better anti-tumor responses in male patients have already been described in the past [24, 25, 30] which makes it necessary to evaluate potential influencing factors also in urooncological tumor entities. To the best of our knowledge, this is the first study assessing changes in sex hormones and the influence of baseline sex hormone values on survival in mUC receiving ICI.

Sex dimorphism of anti-tumor immune response and following efficacy of ICI relies on complex regulation and interactions of genes, microbiome composition and sex hormones [31]. The sole role of gonadotropins in UC has not yet been investigated. However, according to literature, E2 increases production of immunoglobulins [32] whereas androgens, including testosterone, have been reported to suppress immune cell activity [33]. Additionally, regulatory T cells increase with high E2 levels [34]. E2 enhances both cell mediated and humoral immune responses [34, 35], as well as secretion of IgG and IgM [33] and was found to directly up-regulate the expression of mediators of B cell survival [36].

In the study, overall responders to ICI had significantly increased FSH levels, combined with a physiologically associated lowered LH/FSH ratio, yet without sex-specific differences. In male therapy responders, FSH significantly increased when the patient received second line ICI. Testosterone in this cohort yet only showed a slight downward trend. Increased FSH values would physiologically lead to an increase in E2 values, which we, interestingly, we did not observe in our cohort. However, the small number of patients could also mask statistical significance. Based on the already described protective effect of E2 on bladder cancer, it can be hypothesized that the gonadotropic precursors also enhance the protective effect of E2.

Our data demonstrate that high LH values and a high LH/FSH ratio at baseline correlated with better PFS and OS in female patients. In male patients a high E2 level was also indicative for better survival with prolonged PFS and OS, also accounting for improved survival of patients when early sex hormone precursors are present. So far, literature has shown divergent results regarding the relationship between sex and response to ICI. Large meta-analysis showed no significant differences in therapy response to ICI including various cancer types [37, 38]. Further analysis revealed survival benefits for male but not female patients [25] undergoing ICI [24, 39]. However, we could not confirm such a trend in our study cohort and the detailed pathophysiological mechanisms remain unclear. Increased expression of PD-1 has been shown to be mediated by E2 [34]. This could result in a positive advantage in immunological and clinical response for women with higher LH and FSH baseline levels as seen in our cohort and could explain better survival in men with higher E2 levels.

Our study brings up certain limitations, primarily the small number of patients decreases the statistical power and these potential clinically relevant effects need to be validated in a larger collective. In addition, due to the low cohort size, effects of other factors impacting on survival such as tumor stage or metastasis status could not be included in the current analysis. It is of note that we only included postmenopausal women and our collective included patients treated with ICI in the first- and second line.

### Perspectives and significance

First clinical data on modulation of sex hormones and gonadotropins during ICI in mUC are presented with this work. A significant increase in FSH in ICI responders was observed in the overall cohort, as well as in solely male individuals, but not in females. LH/FSH ratio at baseline was increased in female therapy responders. Increased baseline LH and LH/FSH ratio in females and increased baseline E2 in males correlated with better PFS and OS in our cohort. Further investigation of sex hormones and their association on efficacy of ICI is of utter importance to future research to provide more insights in these regulating mechanisms. To date, it is becoming apparent that sex hormones may well represent possible prognostic markers; therefore, more intensive observation of these interactions should become focus of larger cohort studies.

## Conclusions

We provide first evidence that sex hormones can influence the response to immune checkpoint inhibitors as well as survival in patients with metastatic urothelial cancer. Thus, elucidating the interaction between sex hormones and immune responses in men and women could improve our understanding of resistance mechanisms to immune checkpoint inhibitors and better select patients who might benefit most from these specific therapies.

## Supplementary Information

**Additional file 1.** Changes in blood levels of luteinizing hormone, follicle-stimulating hormone, LH to FSH ratio, prolactin, estrogen and testosterone prior to the therapy start, after 6/8 weeks and 12/14 weeks after the therapy begin in females and males. Statistical significance was assessed by Friedman test with Kendall’s W effect size statistic. Points represent single observations. Gray lines connect observations belonging to the same participant. Numbers of complete observations are displayed in the plot captions. Effect size and p values are presented in the plot facets. Female, male.

## Data Availability

Please contact author for data requests.

## Abbreviations

AR: Androgen receptor
UC: Urothelial cancer
CR: Complete remission
E2: Estrogen
EMA: European Medicines Agency
FDA: Food and Drug Administration
FSH: Follicle-stimulating hormone
FU: Follow-up
LH: Luteinizing hormone
OS: Overall survival
ORR: Objective response rate
PD: Progressive disease
PD-1: Programmed cell death protein 1
PD-L1: Programmed cell death 1 ligand 1
PFS: Progression-free survival
PR: Partial remission
SD: Stable disease
