# Supplementary material for: Sex hormones influence survival of patients with metastatic urothelial carcinoma undergoing immune checkpoint therapy
Source: Biol Sex Differ. 2023 Jun 5;14:38. doi: 10.1186/s13293-023-00522-x (PMC10243034; doi:10.1186/s13293-023-00522-x)

Additional Files

Additional File 1. Changes in blood levels of luteinizing hormone (LH), follicle-stimulating hormone (FSH), LH to FSH ratio (LH/FSH), prolactin, estrogen and testosterone prior to the therapy start, after 6/8 weeks and 12/14 weeks after the therapy begin in females and males. Statistical significance was assessed by Friedman test with Kendall's W effect size statistic. Points represent single observations. Gray lines connect observations belonging to the same participant. Numbers of complete observations are displayed in the plot captions. Effect size and p values are presented in the plot facets. Female (n = 10), male (n= 18).

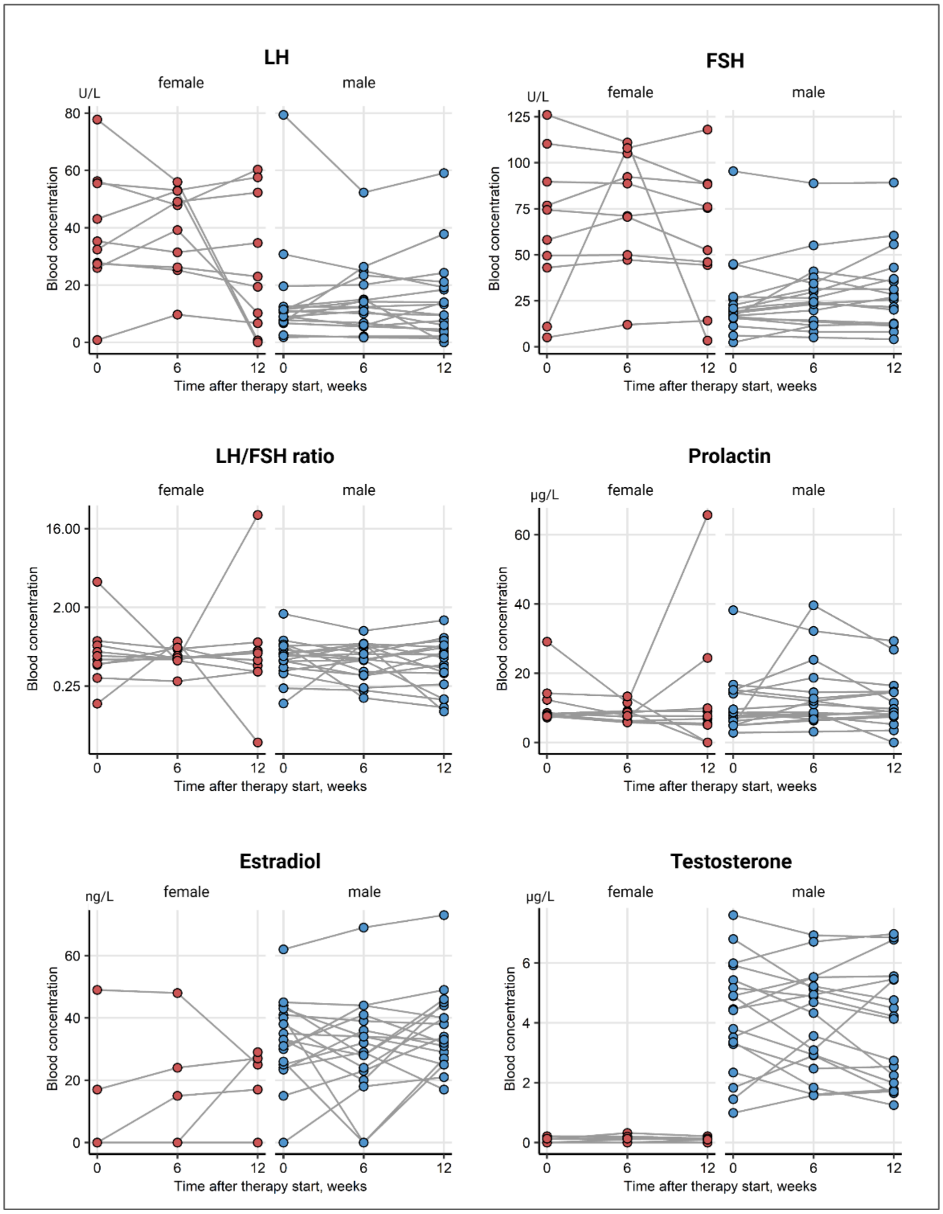

Supplement: Supplementary file 1 — Additional file 1. Changes in blood levels of luteinizing hormone, follicle-stimulating hormone, LH to FSH ratio, prolactin, estrogen and testosterone prior to the therapy start, after 6/8 weeks and 12/14 weeks after the therapy begin in females and males. Statistical significance was assessed by Friedman test with Kendall’s W effect size statistic. Points represent single observations. Gray lines connect observations belonging to the same participant. Numbers of complete observations are displayed in the plot captions. Effect size and p values are presented in the plot facets. Female, male. [file 13293_2023_522_MOESM1_ESM.pdf]
